# Comparative Study of Microstructure and Mechanical Properties of Two TiAl-Based Alloys Reinforced with Carbide Particles

**DOI:** 10.3390/molecules25153423

**Published:** 2020-07-28

**Authors:** Juraj Lapin, Kateryna Kamyshnykova, Alena Klimova

**Affiliations:** Institute of Materials and Machine Mechanics, Slovak Academy of Sciences, Dúbravská cesta 9, 84513 Bratislava, Slovak Republic; kateryna.kamyshnykova@savba.sk (K.K.); alena.klimova@savba.sk (A.K.)

**Keywords:** intermetallics, TiAl, carbides, heat treatment, microstructure, hardness, creep

## Abstract

Microstructure and mechanical properties of two TiAl-based alloys with nominal composition Ti-42.6Al-8.7Nb-0.3Ta-2.0C and Ti-41.0Al-8.7Nb-0.3Ta-3.6C (in at.%) were investigated and compared. The alloys were prepared by vacuum induction melting, followed by centrifugal casting. The as-cast samples were subjected to hot isostatic pressing and heat treatment consisting of solution annealing in β (Ti-based solid solution) phase field, cooling at a constant rate and stabilization annealing. The microstructure of the alloys consists of α_2_ (Ti_3_Al) + γ (TiAl) lamellar grains, single γ phase, coarse Ti_2_AlC particles, and irregular shaped α_2_ phase. The increase in the content of C at the expense of decreasing Al in the studied alloys affects solid-state phase transformation temperatures and leads to a decrease in size of grains and primary Ti_2_AlC particles, increase in the volume fraction of reinforcing carbide particles, decrease in the volume fraction of lamellar colonies, and widening of the grain boundaries. Long-term ageing at 800 °C has no effect on the grain size but leads to the formation of Ti_4_Al_3_Nb particles and increase in interlamellar spacing. The Vickers hardness, microhardness of lamellar grains, indentation nanohardness, and elastic modulus of the boundary γ phase decrease during ageing. The Ti-42.6Al-8.7Nb-0.3Ta-2.0C alloy shows improved creep resistance compared to that of Ti-41.0Al-8.7Nb-0.3Ta-3.6C and some reference TiAl-based alloys at a temperature of 800 °C and applied stress of 200 MPa.

## 1. Introduction

Lightweight intermetallic alloys based on ternary TiAl-Nb system are of great interest for applications in the aerospace, power engineering, and automotive industries due to their high specific strength, high melting temperature, good high temperature creep strength, and oxidation resistance [1,2]. The increasing demand for higher efficiency of aircraft engines and higher operating temperatures of turbocharger wheels enforce the development of more creep-resistant lightweight alloys. Depending on chemical composition and applied processing techniques, TiAl-based alloys can be produced with different types of microstructure such as fully lamellar, nearly lamellar, duplex, and near gamma [3,4,5]. The duplex and near gamma alloys are characterized by higher room temperature ductility, tensile strength, and longer fatigue life than fully or nearly lamellar ones [6]. Better creep resistance of fully or nearly lamellar TiAl-based alloys has been related to highly anisotropic lath structure and reduction in interlamellar spacing [7,8].

It is well known that substitutional elements like Nb, Mo, Ta, and W as well as the interstitial elements such as C improve high temperature creep resistance of TiAl-based alloys [9,10,11,12,13]. The addition of carbon up to about 0.8 at.% contributes to precipitation strengthening through the formation of two types of fine carbides: cubic perovskite-type Ti_3_AlC (P-type) with needle-like morphology and hexagonal Ti_2_AlC (H-type) with plate-like morphology [12,14,15,16]. Higher content of carbon (above 1 at.%) leads to the formation of coarse primary H-type carbide particles during solidification [17,18,19,20,21]. The H-Ti_2_AlC is a thermodynamically stable phase with a unique combination of both metallic and ceramic properties such as high fracture resistance, excellent damage tolerance, good thermal and electrical conductivity, easy machinability, good thermal shock and oxidation resistance, high elastic modulus, and thermomechanical stability [22]. Furthermore, the density and thermal expansion coefficient of Ti_2_AlC are close to those of TiAl-based alloys, which largely avoid the phenomena of segregation of the carbide particles and reduce inner stresses between the reinforcement and the matrix during fabrication. The contribution of carbides to the strengthening of TiAl-based alloys strongly depends on their size and distribution in the intermetallic matrix [23,24,25]. Coarse uniformly distributed primary Ti_2_AlC particles increase toughness by bridging and blunting of propagating cracks [19,26]. Fine secondary P-Ti_3_AlC and H-Ti_2_AlC precipitates improve high temperature creep resistance by hindering dislocation motion and grain boundary sliding [27,28,29]. Despite previous studies on the design and properties of TiAl-based alloys reinforced with carbon particles, very limited information has been published about the effect of substitution of C for Al in a system with fully lamellar structure on solid-state phase transformations, microstructure, distribution of primary carbide particles, and mechanical properties.

This study aims to investigate and compare the microstructure and some mechanical properties of two TiAl-based alloys with nominal composition Ti-42.6Al-8.7Nb-0.3Ta-2.0C and its derivative Ti-41.0Al-8.7Nb-0.3Ta-3.6C (in at.%). The alloys differ in the content of C, which was increased in the derivative alloy at the expense of a decrease in the content of Al. The effect of alloying on microstructure formation during solid-state phase transformations, microstructure stability during long-term ageing, hardness evolution, and creep behavior are reported and discussed.

## 2. Results and Discussion

### 2.1. Solid-State Phase Transformations

**Figure 1:**
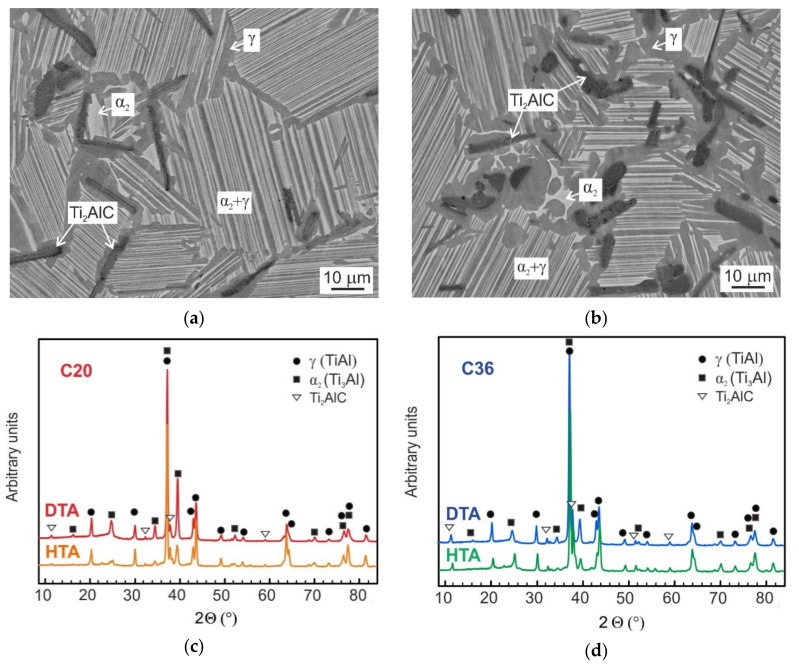
Microstructure and phase composition of DTA samples from C20 and C36 alloys after cooling from a temperature of 1450 °C at a rate of 15 °C/min: (**a**) BSE micrograph showing the microstructure of C20 alloy; (**b**) BSE micrograph showing the microstructure of C36 alloy; (**c**) The typical XRD patterns of C20 alloy; (**d**) The typical XRD patterns of C36 alloy.

The alloys with nominal composition Ti-42.6Al-8.7Nb-0.3Ta-2.0C and Ti-41.0Al-8.7Nb-0.3Ta-3.6C (in at.%) designated as C20 and C36, respectively, were prepared by vacuum induction melting in graphite crucibles followed by a centrifugal casting into a graphite mold. The as-cast alloys were subjected to hot isostatic pressing (HIP) at a temperature of 1260 °C to remove casting porosity. Table 1 summarizes the measured chemical composition of the studied alloys. The alloy C36 differs from the alloy C20 in the content of C, which is increased from 2.0 to 3.6 at.% mainly at the expense of a decrease in the content of Al from 42.6 to 41.1 at.%. The measured content of impurities such as oxygen and nitrogen do not exceed 800 wt.ppm and 100 wt.ppm, respectively. Figure 1a,b show the typical microstructure of differential thermal analysis (DTA) samples after cooling from a temperature of 1450 °C at a constant rate of 15 °C/min. Figure 1c,d show the X-ray diffraction (XRD) patterns of the DTA samples indicating the presence of three coexisting phases: γ-TiAl (tetragonal crystal structure, L1_0_), α_2_-Ti_3_Al (ordered hexagonal crystal structure, D0_19_), and H-Ti_2_AlC (hexagonal crystal structure, Pearson symbol hP8). Chemically, three different phases and two regions (lamellar and grain boundaries) can be identified in the microstructure of the studied alloys, as seen in Figure 1. Table 2 summarizes the measured chemical composition of the coexisting phases in the C20 and C36 alloys.

Based on the measured chemical composition and XRD analyses, the microstructure of the C20 alloy consists of equiaxed α_2_ + γ lamellar grains and grain boundaries containing single γ phase, primary plate-like Ti_2_AlC particles and irregular shaped α_2_ phase, as seen in Figure 1a. The microstructure of the C36 alloy consists of equiaxed α_2_ + γ lamellar grains and wide grain boundaries containing γ phase, coarse Ti_2_AlC particles, and irregular shaped α_2_ phase, as shown in Figure 1b. Table 2 indicates that Nb substitutes partially Ti atoms in Ti_2_AlC phase but its solubility reaches only (0.69 ± 0.03) of the average content of Nb in the studied alloys. This value is lower than that of (0.78 ± 0.06) reported by Klimova and Lapin [24] for intermetallic Ti-Al-Nb-C-Mo alloys reinforced with coarse primary Ti_2_AlC particles. On the other hand, the solubility of Ta in Ti_2_AlC is high and reaches (1.95 ± 0.03) of the average content of Ta in the C20 and C36 alloys. It should be mentioned that the morphology of the coarse primary Ti_2_AlC particles depends strongly on the content of C. While the C20 alloy contains only long plate-like primary carbides, the C36 alloy contains both plate-like and numerous irregular shaped carbide particles formed preferentially along the lamellar grain boundaries, as seen in Figure 1a,b. Both plate-like and irregular shaped Ti_2_AlC particles are formed during solidification of the studied alloys according to the transformation pathway L (liquid) + TiC (face-centered cubic crystal structure, Pearson symbol cF8) → L + Ti_2_AlC [18,30]. This reaction/transformation starts by the growth of solid Ti_2_AlC layer at the L/TiC interfaces and leads to either full transformation of small TiC particles to Ti_2_AlC or the formation of continuous Ti_2_AlC layer around TiC clusters preserving the TiC phase in the cores of some irregular shaped carbide particles during fast cooling [17]. The retained TiC phase is unstable and transforms to the thermodynamically stable Ti_2_AlC phase during solution annealing in α (Ti-based solid solution with the hexagonal crystal structure, A3) or α + β (Ti-based solid solution with the cubic crystal structure, A2) phase fields [18,24].

**Figure 2:**
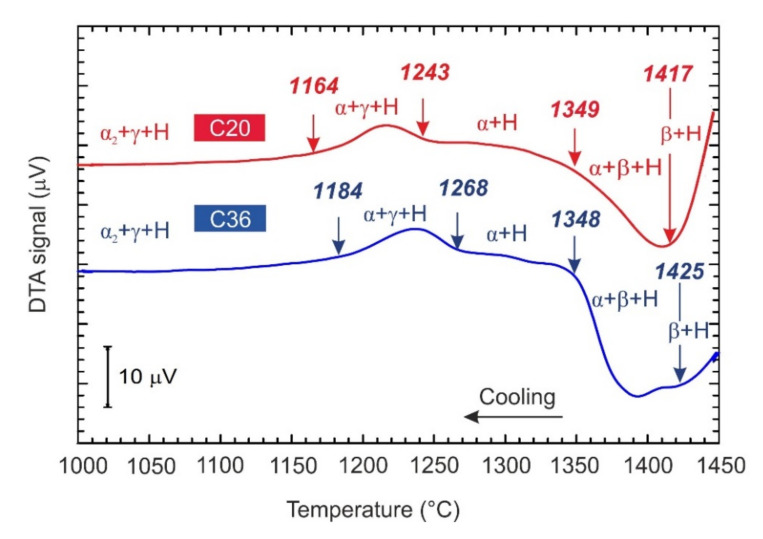
The typical DTA cooling curves of the studied C20 and C36 alloys.

**Figure 3:**
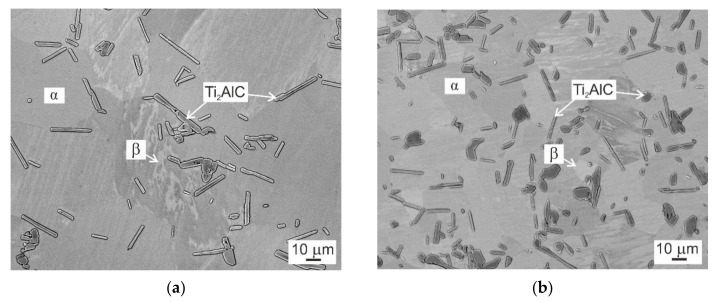
BSE micrographs showing the microstructure of the studied alloys after water quenching from a temperature of 1400 °C: (**a**) C20 alloy; (**b**) C36 alloy.

Figure 2 shows the typical DTA curves of the C20 and C36 alloys obtained during cooling from a temperature of 1450 °C at a constant cooling rate of 15 °C/min. The DTA cooling curves, which are shifted vertically for clarity, indicate exothermic transformations. Two onset temperatures of the exothermic transformation represent the start and finish of the β transformation to α phase according to phase transformation sequence β + H → β + α + H → α + H. The onset start transformation temperature is slightly affected by the content of C and Al and increase from 1417 to 1425 °C with the increasing content of C from 2.0 to 3.6 at.% and decreasing content of Al from 42.6 to 41.1 at.%. The onset finish transformation temperature is not affected by the alloying and corresponds to 1349 and 1348 °C for the C20 and C36 alloy, respectively. As has been reported by several authors [31,32], high-temperature β and α phases can be preserved in the microstructure by fast cooling. Figure 3 shows the microstructure of the C20 and C36 alloys after water quenching from a solution annealing temperature of 1400 °C, which is below the β to α phase start transformation temperature of 1417 and 1425 °C in the C20 or C36 alloy, respectively. Three phases can be identified in the microstructure of the quenched samples: α (dark grey color phase), β (white grey color phase), and Ti_2_AlC particles confirming β + α + H phase field for both studied alloys at 1400 °C. The onset temperatures of 1243 and 1164 °C and a 1268 and 1184 °C represent the start and finish of the α phase decomposition according to phase transformation sequence α + H → α + γ+ H for the C20 and C36 alloy, respectively. Both the onset start and finish α phase decomposition temperatures are affected by the content of C and Al and are lowe*r* for the C20 alloy compared to those of the C36 one. Below the finish α phase decomposition temperature, the phase transformation sequence α + γ + H → α_2_ + γ+ H is identified in the studied alloys by XRD analysis, as seen in Figure 1c,d.

### 2.2. Effect of Heat Treatment on Microstructure

**Figure 4:**
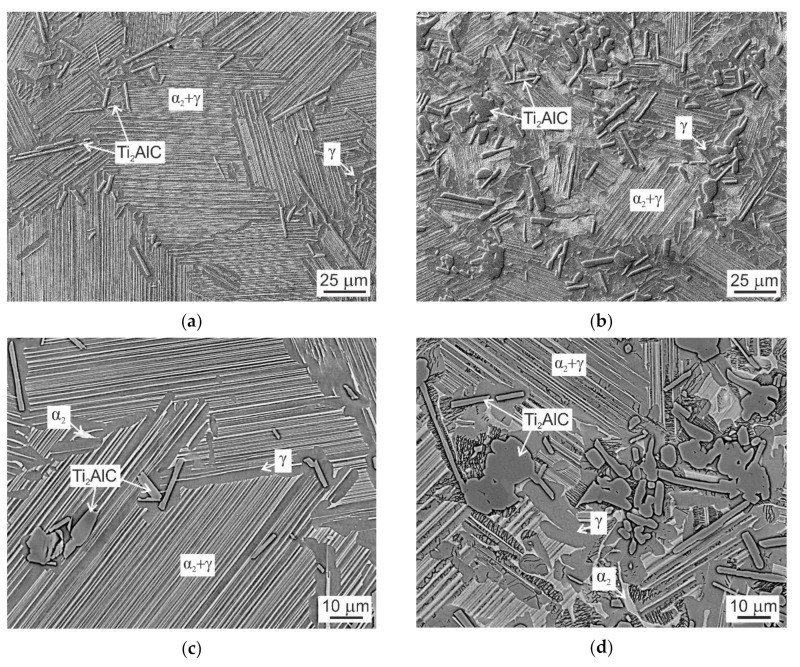
Micrographs showing the microstructure of C20 and C36 alloys after heat treatment: (**a**) Lamellar grain structure of HT C20 alloy, SEM; (**b**) Grain structure of HT C36 alloy, SEM; (**c**) Grain boundaries with γ phase, primary Ti_2_AlC particles and α_2_ phase in HT C20 alloy, BSE; (**d**) Grain boundaries with γ phase, primary Ti_2_AlC particles and α_2_ phase in HT C36 alloy, BSE.

**Figure 5:**
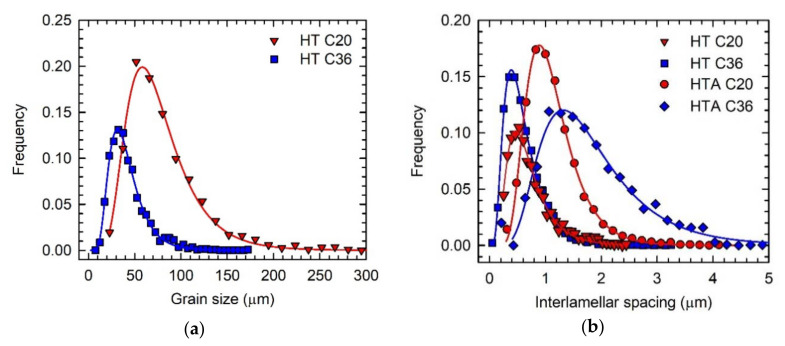
Measured grain size and interlamellar spacing in C20 and C36 alloys after heat treatment and heat treatment combined with ageing: (**a**) The typical log-normal distribution curves of grain size; (**b**) The typical log-normal distribution curves of α_2_-α_2_ interlamellar spacing. The types of distribution curves are marked in the figures.

The C20 and C36 alloys used for the evaluation of microstructural stability and mechanical properties were subjected to the heat treatment after HIP-ing. The heat treatment consisted of solution annealing in the single β phase field at a temperature of 1460 °C followed by cooling at a constant cooling rate of 15 °C/min and stabilization annealing at 850 °C for 25 h. Several authors [12,15,16,19,27,33] have shown that the stabilisation annealing in a temperature range from 800 to 900 °C leads to the formation of fine secondary P-Ti_3_AlC and H-Ti_2_AlC precipitates along α_2_/γ lamellar interfaces, which significantly improve the high temperature creep resistance of carbon-containing TiAl-based alloys. Figure 4 shows the typical microstructure of the heat-treated (HT) alloys. The microstructure of the HT C20 and HT C38 consists of fully lamellar α_2_ + γ grains separated by γ grain boundaries containing Ti_2_AlC particles and irregular shaped α_2_ phase, as seen in Figure 4a–d. It should be noted that the chemical compositions of the coexisting phases and regions measured in the HT samples correspond to those of DTA samples (Table 2) and all deviations fall only within the experimental error of measurements. To compare the microstructure of the HT C20 and HT C36 alloys, microstructural parameters such as grain size, α_2_-α_2_ interlamellar spacing, size of primary carbide particles and volume fractions of lamellar grains, grain boundaries and primary carbide particles are measured and experimental data are evaluated statistically. Figure 5a shows that the measured data of grain size d (more than 1000 measurements) can be fitted by a log-normal distribution function. The statistical analysis leads to a mean grain size of d = 70.6 ± 0.8 µm for the HT C20 alloy, which is a significantly higher value than that of d = 37.7 ± 0.6 µm measured for the HT C36 alloy. The statistical analysis of the measured α_2_-α_2_ interlamellar spacing λ of the studied alloys (more than 2200 measurements for each alloy) shows that the best fit can be achieved by a log-normal distribution function, as seen in Figure 5b. The log-normal distribution curves result in a mean α_2_-α_2_ interlamellar spacing of λ = 0.62 ± 0.01 µm for the HT C20 alloy, which is a higher value than that of λ = (0.54 ± 0.01) µm measured for the HT C36 alloy. Table 3 summarises the measured grain size d, α_2_-α_2_ interlamellar spacing λ, length of the major axis of Ti_2_AlC particles L, volume fractions of lamellar grains V_lg_ and grain boundaries V_gb_, the volume fraction of primary Ti_2_AlC particles formed along the grain boundaries V_Cgb_, and average volume fraction of Ti_2_AlC particles in the HT C20 and HT C36 alloys V_C_. The increase in the content of C at the expense of the decrease in the content of Al in the HT C36 alloy leads to a significant decrease in the volume fraction of lamellar grains to V_lg_ = 48 vol.% and increase in the volume fraction of grain boundaries to V_gb_ = 52 vol.% compared to those of 72 and 28 vol.%, respectively, in the HT C20 alloy. The higher content of C leads to the formation of a higher volume fraction of the primary Ti_2_AlC particles of V_C_ = 14.1 vol.%, which are preferentially distributed along the γ grain boundaries achieving V_Cgb_ = 13.9 vol.% in the HT C36 alloy compared to those of V_C_ = 4.1 vol.% and V_Cgb_ = 3.4 vol.%, respectively, measured in the HT C20 alloy. The formation of higher volume fraction of the primary Ti_2_AlC particles in the HT C36 alloy leads to a decrease in mean length of their major axis to L = 9.5 μm compared to that of L = 10.8 μm measured in the HT C20 alloy. Despite decreasing content of Al, the increasing content of C in the HT C36 alloy leads to the formation of wider grain boundaries composed of a higher volume fraction of the γ phase (38.1 vol.%) and primary Ti_2_AlC particles (13.9 vol.%) compared to the grain boundaries of the C20 alloy containing 24.6 vol.% of γ phase and 3.4 vol.% of Ti_2_AlC particles.

### 2.3. Effect of Ageing on Microstructure Stability

**Figure 6:**
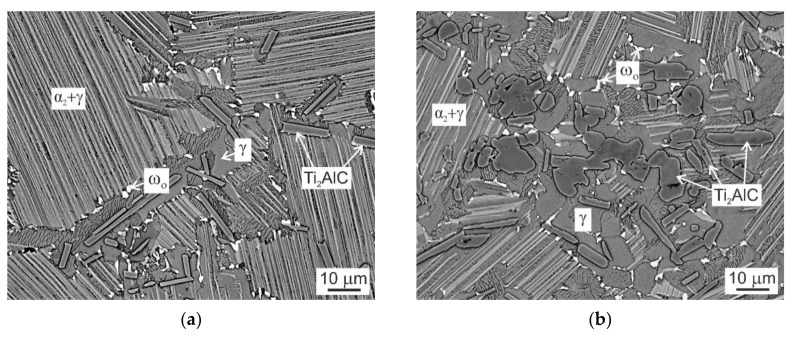
BSE micrographs showing the microstructure of C20 and C36 alloys after heat treatment combined with ageing: (**a**) HTA C20 alloy; (**b**) HTA C36 alloy. The coexisting phases and regions are marked in the figures.

Figure 6 shows the typical microstructure of the C20 and C36 alloys after ageing at 800 °C for 1240 h. The microstructure of the heat-treated and aged (HTA) alloys consists of equiaxed α_2_ + γ lamellar grains and grain boundaries containing single γ phase reinforced with coarse primary Ti_2_AlC particles and a small amount of white color phase formed at the grain boundaries. However, the XRD patterns of the HTA alloys show the presence of only three phases: α_2_, γ and Ti_2_AlC, as seen Figure 1c,d. Table 4 summarizes the measured chemical composition of the coexisting phases and regions in the HTA alloys. Taking into account the measured chemical composition, temperature of the formation and assuming experimental study of ternary Ti-Al-Nb systems and more complex Ti-45Al-8.5Nb-0.2W-0.2B-0.02Y (at.%) alloy reported by Witusiewicz et al. [34] and Wang et al. [35], respectively, the white color particles can be related to Ti_4_NbAl_3_ phase (hexagonal crystal structure, B8_2_). The chemical composition of the coexisting phases in the HT (see Table 2) and HTA (see Table 4) alloys are slightly different. The main difference can be seen in the redistribution of Nb, whose content is increased in the ω_o_-Ti_4_NbAl_3_ particles at the expense of decreasing its amount in the γ phase and α_2_ + γ lamellar regions. Since Nb is a low diffusing element in TiAl characterized by a diffusion coefficient of about D = 4.56 × 10^−19^ m^2^/s at 800 °C [36], long-term ageing is required for the formation of ω_o_ particles. Figure 1 and Figure 4 clearly show that the ω_o_ particles are not formed in the studied alloys during continuous cooling from the solution annealing temperature or during stabilization annealing at a temperature of 850 °C for 25 h. The present results are in a good agreement with the experimental study and thermodynamic calculations of Witusiewicz et al. [34] showing that the Ti_4_NbAl_3_ is a stable phase at a temperature of 800 °C and decomposes according to a reaction Ti_4_NbAl_3_ ↔ α_2_ + γ at a temperature higher than 810 °C.

Table 3 indicates that the long-term ageing at a temperature of 800 °C for 1240 h has no effect on the mean grain size d, mean length of major axis L and average volume fraction of the primary Ti_2_AlC particles V_C_ compared to those measured in the HT alloys. However, the mean interlamellar spacing λ (see Figure 5b) increases significantly and the volume fraction of lamellar grains decreases in the HTA alloys compared to those in the HT ones, as summarized in Table 3. The increase in the α_2_-α_2_ interlamellar spacing can be attributed to the diffusion-controlled lateral growth of the γ lamellae at the expense of dissolution of α_2_ lamellae [37,38]. In carbon-containing TiAl-based alloys, the widening of γ lamellae is hindered by the carbon atoms, which segregate to ledges and kinks at α_2_/γ lamellar interfaces and form fine secondary P-Ti_3_AlC or H-Ti_2_AlC precipitates [27,39].

### 2.4. Vickers Hardness and Elastic Modulus

**Figure 7:**
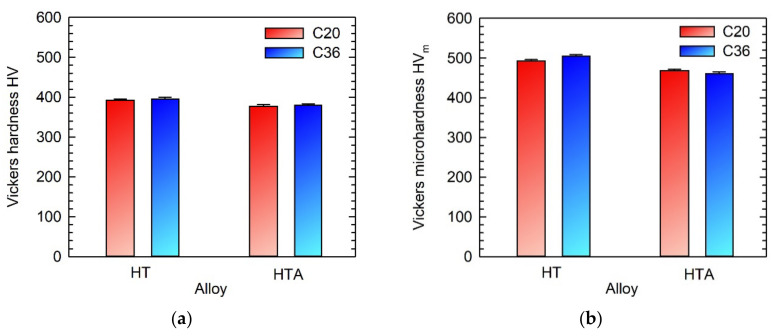
(**a**) Vickers hardness HV and (**b**) Vickers microhardness of lamellar grains HV_m_ of C20 and C36 alloys after heat treatment and heat treatment combined with ageing. The type of alloys and applied treatments are marked in the figures.

Figure 7 shows Vickers hardness HV and Vickers microhardness of lamellar grains HV_m_ of the C20 and C36 alloys after heat treatment and heat treatment combined with ageing. The increase in the content of C and decrease in the content of Al in the HT C36 alloy have no significant effect on the HV values compared to that of HT C20 alloy and all deviations fall into the error of measurements. The long-term ageing leads to a small decrease in HV values by about 4% but no statistical differences can be found between HT C20 and HTA C36 samples, as shown in Figure 7a. Figure 7b shows Vickers microhardness HV_m_ of the α_2_ + γ lamellar grains. The microhardness of the HT C36 sample is higher than that of the HT C20 one, which can be explained by a finer lamellar structure of the C36 alloy (see Table 3). As shown by Lapin [40], Vickers microhardness HV_m_ increases with decreasing interlamellar spacing λ according to the relationship $HV_{m}\propto 1/\sqrt{\lambda }$. The long-term ageing leads to a decrease of HV_m_ values in both HTA C20 and HTA C36 alloys, which can be related to an increase in interlamellar spacing λ (see Table 3).

**Figure 8:**
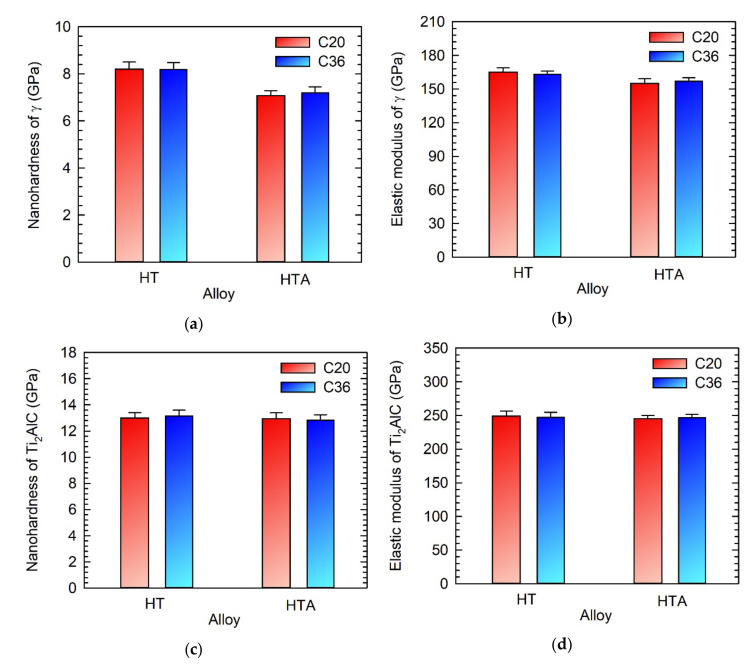
Indentation nanonahardness and elastic modulus of C20 and C36 alloys after heat treatment and heat treatment combined with ageing: (**a**) Indentation nanohardness of grain boundary γ phase; (**b**) Indentation elastic modulus of grain boundary γ phase; (**c**) Indentation nanohardness of primary Ti_2_AlC particles; (**d**) Indentation elastic modulus of primary Ti_2_AlC particles. The type of alloys and applied treatments are marked in the figures.

Figure 8 shows indenation nanohardness and indentation elastic modulus of the γ phase and primary Ti_2_AlC particles formed along the grain boundaries in the C20 and C36 alloys. The increase in the content of C and decrease in the content of Al in the HT C36 alloy have no statistical effect on indentation nanohardness and elastic modulus measured in the γ phase formed along the grain boundaries compared to those of the HT C20 alloy. The nanohardness of the γ phase is evaluated to be (8.2 ± 0.3) GPa in both alloys, which is a comparable value with that of 8.5 GPa but higher than that of 6.8 GPa reported for Ti-43.7Al-4.1Nb-1.1Mo-0.1B-0.78C and Ti-43.5Al-4Nb-1Mo-0.1B (at.%) alloys, respectively [41,42]. The high nanohardness values of the γ phase can be related to its solution hardening by C, whose solubility has been measured to be up to 0.3 at.% [41]. The long-term ageing leads to a decrease of indentation nanohardness and elastic modulus of the boundary γ phase in the HTA C20 and HTA C36 alloys, as seen in Figure 8a,b. The decrease of nanohardness can be attributed to the formation of secondary carbide precipitates and local recovery and/or recrystallization of the γ phase [27]. Figure 8c,d indicate that the chemical composition and applied heat treatment have no statistical effect on the indentation nanohardness and indentation elastic modulus of coarse primary Ti_2_AlC particles. The measured elastic modulus of (247 ± 6) GPa is comparable with the values ranging from 245 to 260 GPa reported for Ti_2_AlC by Velasco et al. [43].

### 2.5. Creep Behaviour

**Figure 9:**
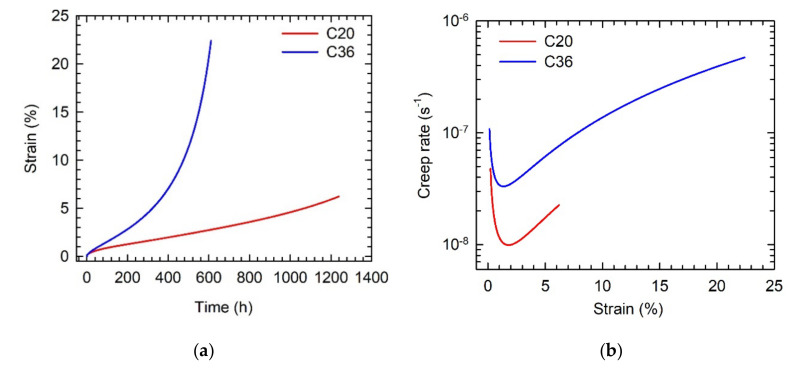
Creep deformation curves of C20 and C36 alloys at a temperature of 800 °C and applied stress of 200 MPa: (**a**) Dependence of creep strain on time; (**b**) Dependence of creep rate on strain. The studied alloys are indicated in the figures.

Figure 9 shows tensile creep deformation curves for the HT alloys at a temperature of 800 °C and applied stress of 200 MPa. While the creep of the C20 alloy was interrupted after 1240 h corresponding to a strain of 6.2%, the C36 alloy was tested up to a creep fracture achieved after 610 h at a strain of 22.4%, as shown in Figure 9a. The creep deformation curves indicate an improved creep resistance of the C20 alloy compared to that of the C36 one. Both creep curves exhibit primary creep stage, which is directly followed by the tertiary creep, as seen in Figure 9b. During the primary creep stage, the creep rate decreases to a minimum value of 9.9 × 10^−9^ s^−1^ at a strain of 1.8% and 3.31 × 10^−8^ s^−1^ at a strain of 1.3% and then increases with increasing strain in the C20 and C36 alloy, respectively. Besides lower minimum creep rate, a significantly improved creep resistance of the C20 alloy can be observed during whole creep deformation. After the creep for 610 h, the creep strain achieves only 2.8% in the C20 alloy compared to that of 22.4% in the C36 one. The tertiary creep stage is characterized by an increase in the creep strain and creep rate and results from the degradation of the initial HT microstructure of the studied alloys.

**Figure 10:**
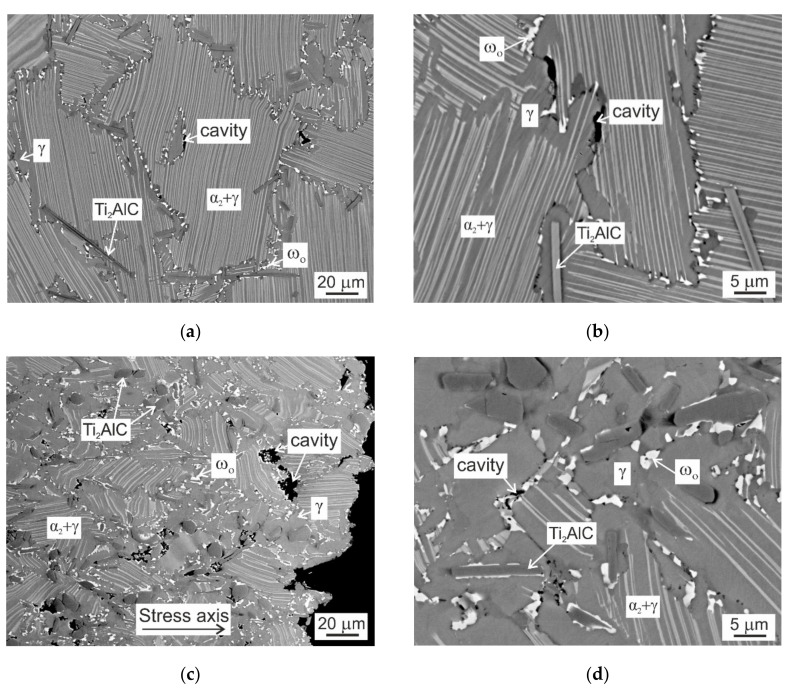
BSE micrographs showing longitudinal sections of C20 and C36 alloys after creep testing at a temperature of 800 °C and applied stress of 200 MPa: (**a**,**b**) Microstructure of the C20 creep specimen tested to a strain of 6.2% for 1240 h; (**c**,**d**) Microstructure of the C36 creep specimen tested to a fracture for 610 h.

Figure 10 shows the typical microstructures of the C20 and C36 creep specimens tested to a strain of 6.2 and 22.4%, respectively. Figure 10a,b indicate that the deformed lamellar grains of the C20 alloy contain relatively stable α_2_ lamellae and the primary plate-like Ti_2_AlC particles are not fragmented and pin effectively the grain boundaries. The main microstructural instabilities can be related to the formation of numerous ω_o_ particles and cavities of various size along the lamellar grain boundaries. The cavitation along the grain boundaries strongly depends on the orientation of the neighboring grains. The soft oriented grains have lamellae inclined at an angle of 35° or 75° and hard oriented grains have lamellae parallel or perpendicular to the stress axis [44]. The cavities formed along the hard/hard oriented grains are usually larger in size than those formed between the soft/hard oriented grains [10]. The creep of the C36 alloy is accompanied by bending and fragmentation of some plate-like and irregular shaped Ti_2_AlC particles, the formation of numerous ω_o_ particles, and widening of the grain boundaries at the expense of decreasing volume fraction of lamellar colonies. The ω_o_ phase is preferentially formed along the grain boundaries and frequently at Ti_2_AlC/matrix interfaces, as shown in Figure 10c,d. The ω_o_ particles formed along the grain boundaries interlock the neighboring grains, reduce effectively grain boundary sliding, and improve fracture resistance by preventing the formation of grain boundary wedge cracks. The formation of the γ + ω_o_ + Ti_2_AlC type of microstructure along the grain boundaries between the soft/hard and hard/hard oriented grains affects significantly the cavitation mechanisms and fracture resistance of the C36 alloy. The deformation occurring continuously in the soft boundary γ phase leads to the nucleation and coalescence of the cavities. The growth of cavities formed along the grain boundaries is controlled by diffusion and strongly affected by the creep induced formation of secondary phases [45,46]. The fracture of the C36 alloy occurs due to overloading with the fracture path propagating preferentially intergranularly, as seen in Figure 10c.

**Figure 11:**
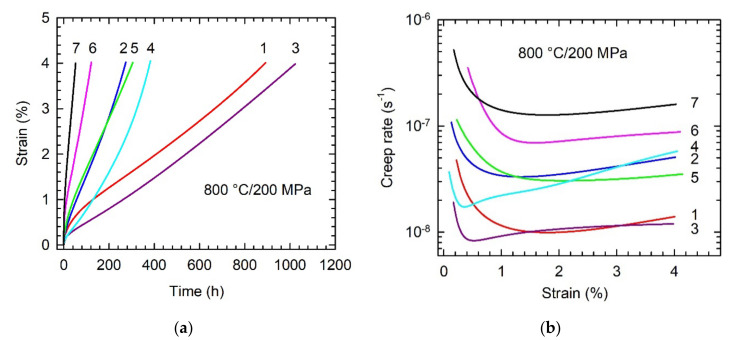
Creep deformation curves at a temperature of 800 °C and applied stress of 200 MPa: (**a**) Dependence of creep strain on time; (**b**) Dependence of creep rate on strain. Designation and composition of reference alloys (in at.%): 1—studied C20 alloy, 2—studied C36 alloy, 3—Ti-46.4Al-5.1Nb-1C-0.2B, 4—Ti-46Al-8Ta, 5—Ti-45Al-2W-0.6Si-0.7B, 6—Ti-46Al-2W-0.5Si and 7—Ti-44.6Al-7.9Nb-3.6C-0.7Mo-0.1B.

Figure 11 shows creep deformation curves of the C20 and C36 alloys compared to those of other TiAl-based alloys tested at a temperature of 800 °C and applied stress of 200 MPa. The studied C20 alloy shows more extended primary creep stage and similar creep resistance up to a strain of 4% compared to those of Ti-46.4Al-5.1Nb-1C-0.2B (at.%) alloy with fully lamellar microstructure reinforced with 2.3 vol.% of coarse primary Ti_2_AlC particles [5], as shown in Figure 11a,b. The creep resistance of the C20 alloy is improved compared to that of the studied C36 alloy or reference alloys (in at.%) such as Ti-45Al-2W-0.6Si-0.7B with nearly lamellar structure [13], Ti-46Al-8Ta with convoluted structure [10,47], Ti-46Al-2W-0.5Si with pseudo-duplex structure [48], and Ti-44.6Al-7.9Nb-3.6C-0.7Mo-0.1B with γ matrix reinforced with 14.6 vol.% of coarse primary Ti_2_AlC particles and fine secondary P-Ti_3_AlC and H-Ti_2_AlC precipitates [27]. The improvement in the creep strength of the C20 alloy can be attributed to the stabilization of the fully lamellar α_2_ + γ structure and its reinforcement with plate-like primary Ti_2_AlC particles as well as with fine secondary P-Ti_3_AlC and H-Ti_2_AlC precipitates forming in carbon-containing TiAl-based alloys during heat treatment and creep exposure [12,15,27,33,49]. The C36 alloy shows comparable creep strength to that of Ti-45Al- 2W-0.6Si-0.7B (at.%) alloy with nearly lamellar structure containing numerous ribbon-like boride particles and Ti_5_Si_3_ precipitates [13]. The improved creep resistance of the C36 alloy compared to Ti-44.6Al-7.9Nb-3.6C-0.7Mo-0.1B (at.%) alloy with single γ phase matrix reinforced with carbide particles [27] can be attributed to the presence of α_2_ + γ lamellar colonies and larger grain size of the studied C36 alloy. As reported by Maruyama et al. [50] or Kim and Kim [51], because of easy dynamic recrystallization, creep rate of fully lamellar TiAl-based alloys is independent of grain size larger than about 100 μm. However, room temperature ductility decreases with increasing grain size [6]. 

## 3. Materials and Methods

The studied TiAl-based alloys with designed nominal composition Ti-42.6Al-8.7Nb-0.3Ta-2.0C and Ti-41.0Al-8.7Nb-0.3Ta-3.6C (at.%) were prepared by vacuum induction melting of pure metals (purity 99.99%) and addition of graphite powder in graphite crucibles and centrifugally cast into graphite mold using the procedure described elsewhere [18,52]. The conical as-cast samples with a minimum diameter of 12 mm, maximum diameter of 15 mm and length of 150 mm were subjected to HIP at a temperature of 1260 °C and applied pressure of 200 MPa for 4 h in a protective argon atmosphere. Heat treatment of the HIP-ed samples consisted of solution annealing at a temperature of 1460 °C for 50 min and cooling at a constant rate of 15 °C/min to a temperature of 600 °C and furnace cooling to room temperature under protective argon atmosphere. The heat treatment was finalized by stabilization annealing at a temperature of 850 °C for 25 h in air. During heat treatment, the temperature of the samples was measured by PtRh10-Pt thermocouple (type S, Omega, CT, USA) touching the sample surface. The HT samples were cut transversally to a length of 10 mm and subjected to long-term isothermal ageing at a temperature of 800 °C for 1240 h.

Solid-state phase transformation temperatures were determined by DTA in alumina crucibles using alumina powder as the reference standard. The DTA samples were cut from the HT samples by electro spark machining and lathe machined to a diameter of 6 mm and length of 6 mm. The samples were heated to a temperature of 1450 °C at a heating rate of 15 °C/min, hold at this temperature for 10 min and then cooled to room temperature at a cooling rate of 15 °C/min under protective argon atmosphere. The maximum sample temperature was selected below the onset of the solidus temperatures of the studied alloys to avoid reactions between the melt and alumina crucible [53]. In this study, the phase transformation temperatures were determined only from the cooling DTA curves.

Vickers hardness measurements were carried out at an applied load of 298 N, holding time at the point of load application of 10 s and rate of load application of 10 N/s on HT and HTA samples. Vickers microhardness measurements of lamellar grains were performed at an applied load of 0.49 N and dwell time of 10 s on polished and slightly etched sections of the HT and HTA samples. Instrumented nanoindentation measurements of the coexisting phases were carried out at an applied load of 0.01 N and holding time at the point of load application of 2 s on polished and slightly etched samples using a nanoindenter with Berkovich tip of the indenter.

Cylindrical creep specimens with a gauge diameter of 6 mm and a gauge length of 30 mm were lathe machined from HT samples. Constant load tensile creep tests were carried out at a temperature of 800 °C under an initial stress of 200 MPa in air. The test temperature was monitored with two thermocouples touching the specimen gauge section and held constant within ±1 °C. Elongation was measured using a high-temperature extensometer attached to the ledges of the creep specimen. The extensometer was equipped with a linear variable displacement transformer (LVDT). The acquisition of time-elongation data was accomplished by a computer and data processing was performed by a computer program.

Metallographic preparation of the samples consisted of standard grinding using abrasive papers, polishing on diamond pastes with various grain size up 1 µm and etching in a solution of 100 mL H_2_O, 6 mL HNO_3_, and 3 mL HF. Microstructure evaluation was performed by scanning electron microscopy in secondary electron (SEM) and back-scattered electron (BSE) modes using JEOL JSM-7600F microscope (JEOL, Tokyo, Japan). The chemical composition of the samples was evaluated by energy-dispersive spectrometry (EDS). The EDS system applied for measurements of chemical compositions of carbide particles was calibrated using standards. The average oxygen and nitrogen content was measured by LECO ONH836 elemental analyzer. The average carbon content was determined by LECO CS844 elemental analyzer. X-ray diffraction (XRD) analysis was carried out by a Bruker D8 Discover diffractometer (Billerica, MA, USA) equipped with X-ray tube with rotating Cu anode operating at 12 kW. The volume fraction of phases, grain size, size of carbide particles, and interlamellar spacing were measured by computerized image analysis using digitalized micrographs and the measured data were treated statistically.

## 4. Conclusions

The microstructure and mechanical properties of two TiAl-based alloys with nominal composition Ti-42.6Al-8.7Nb-0.3Ta-2.0C and Ti-41.0Al-8.7Nb-0.3Ta-3.6C (in at.%) designated as C20 and C36, respectively, were investigated and compared. The following conclusions are reached.

1. The microstructure of the studied C20 and C36 alloys consists of equiaxed α_2_ + γ lamellar grains, single γ phase, coarse primary Ti_2_AlC particles, and small amount of irregular shaped α_2_ phase. The increase in the content of C on the expense of the decreasing content of Al in the C36 alloy compared to that in the C20 alloy affects solid-state phase transformations and leads to an increase of the start β → α and the start and finish α → α + γ phase transformation temperatures.

2. The increase in the content of C at the expense of decreasing content of Al in the HT C36 alloy leads to an increase in the volume fraction and decrease in size of the primary Ti_2_AlC particles, decrease in grain size, and decrease in the volume fraction of α_2_ + γ lamellar colonies compared to those measured in the HT C20 alloy.

3. The long-term ageing at 800 °C for 1200 h has no statistical effect on the grain size and size and volume fraction of the primary Ti_2_AlC particles. The ageing leads to a significant increase in α_2_-α_2_ interlamellar spacing and decrease in the volume fraction of lamellar colonies in both HTA alloys compared to those of HT ones. The ageing is accompanied by the formation of Nb-rich particles along grain boundaries with the chemical composition corresponding to ω_o_-Ti_4_Al_3_Nb phase.

4. The variation in the content of C and Al has no statistical effect on Vickers hardness of the HT C20 and HT C36 alloys. The Vickers hardness of the HTA alloys decreases compared to that of HT ones but no statistical differences are found between the C20 and C36 alloy. The Vickers microhardness of the lamellar grains is slightly lower in the HT C20 alloy compared to that in the HT C36 one. The long-term ageing of the HTA alloys leads to a significant decrease in Vickers microhardness of lamellar grains compared to that of the HT ones. The measured indentation nanohardnes and elastic modulus of the boundary γ phase are affected by the applied treatment but no statistical differences can be identified between the studied alloys.

5. The creep resistance of the C20 alloy with nearly lamellar structure reinforced with a low volume fraction of primary Ti_2_AlC particles is improved compared to that of C36 alloy and reference TiAl-based alloys with fully lamellar, nearly lamellar, convoluted, pseudo-duplex structures, and the alloy with γ matrix reinforced with carbide particles.

## Figures and Tables

**Table 1:** Chemical composition of studied alloys (at.%) and content of O and N (wt.ppm).

Alloy	Ti	Al	Nb	Ta	C	O	N
**C20**	46.4 ± 0.4	42.6 ± 0.4	8.7 ± 0.1	0.3 ± 0.1	2.0 ± 0.1	775 ± 25	84 ± 15
**C36**	46.3 ± 0.3	41.1 ± 0.4	8.7 ± 0.1	0.3 ± 0.1	3.6 ± 0.1	760 ± 30	76 ± 15

**Table 2:** Chemical composition of coexisting phases and regions of the studied alloys (at.%).

Alloy	Phases	Ti	Al	Nb	Ta	C
**C20**	γ	44.5 ± 0.3	46.7 ± 04	8.7 ± 0.1	0.10 ± 0.05	-
	α_2_	55.1 ± 0.3	35.6 ± 0.4	9.2 ± 0.2	0.10 ± 0.05	-
	α_2_ + γ	47.1 ± 0.3	43.8 ± 0.4	9.0 ± 0.2	0.10 ± 0.05	-
	Ti_2_AlC	40.8 ± 0.5	24.7 ± 0.5	5.9 ± 0.2	0.60 ± 0.10	28.0 ± 0.4
**C36**	γ	44.5 ± 0.3	46.9 ± 0.4	8.5 ± 0.2	0.10 ± 0.05	-
	α_2_	54.6 ± 0.4	35.9 ± 0.4	9.3 ± 0.2	0.20 ± 0.05	-
	α_2_ + γ	47.0 ± 0.3	43.9 ± 0.4	9.0 ± 0.2	0.10 ± 0.05	-
	Ti_2_AlC	41.5 ± 0.4	24.5 ± 0.4	6.1 ± 0.2	0.60 ± 0.10	27.3 ± 0.4

**Table 3:** Measured microstructural parameters of C20 and C36 alloys after heat treatment and heat treatment combined with ageing.

Alloy		Grain Size d (μm)	Interlamellar Spacing λ (μm)	Major Axis of Carbides L (μm)	Volume Fraction V_lg_/V_gb_ (vol.%)	Volume Fraction V_Cgb_ (vol.%)	Average Volume Fraction V_C_ (vol.%)
**C20**	HT	70.6 ± 0.8	0.62 ± 0.02	10.8 ± 0.2	72/28	3.4 ± 0.2	4.1 ± 0.2
	HTA	69.2 ± 0.9	1.04 ± 0.02	10.7 ± 0.2	68/32	3.7 ± 0.2	4.2 ± 0.2
**C36**	HT	37.7 ± 0.6	0.54 ± 0.03	9.5 ± 0.4	48/52	13.9 ± 0.4	14.1 ± 0.4
	HTA	36.4 ± 0.9	1.66 ± 0.03	9.7 ± 0.4	39/61	14.0 ± 0.4	14.2 ± 0.4

**Table 4:** Chemical composition of coexisting phases in HTA C20 and HTA C36 alloys (at.%).

Alloy	Phases	Ti	Al	Nb	Ta	C
**HTA C20**	γ	44.6 ± 0.3	46.9 ± 0.3	8.5 ± 0.2	0.10 ± 0.05	-
	ω_o_	50.5 ± 0.3	37.0 ± 0.3	12.3 ± 0.2	0.20 ± 0.10	-
	α_2_ + γ	47.1 ± 0.3	44.1 ± 0.4	8.7 ± 0.3	0.10 ± 0.05	-
	Ti_2_AlC	40.6 ± 0.4	24.7 ± 0.6	5.9 ± 0.2	0.60 ± 0.10	28.2 ± 0.4
**HTA C36**	γ	44.4 ± 0.3	46.9 ± 0.4	8.6 ± 0.2	0.10 ± 0.05	-
	ω_o_	50.4 ± 0.3	37.1 ± 0.4	12.3 ± 0.2	0.20 ± 0.10	-
	α_2_ + γ	47.0 ± 0.3	44.2 ± 0.4	8.7 ± 0.3	0.10 ± 0.05	-
	Ti_2_AlC	40.8 ± 0.4	24.6 ± 0.5	6.0 ± 0.2	0.60 ± 0.10	28.0 ± 0.5

## References

[1] Kim Y.W., Kim S.L. (2018). Advances in gammalloy materials-processes-application technology: Successes, dilemmas, and future. JOM.

[2] Bewlay B.P., Nag S., Suzuki A., Weimer M.J. (2016). TiAl alloys in commercial aircraft engines. Mater. High. Temp..

[3] Appel F., Paul J.D.H., Oehring M. (2011). Gamma Titanium Aluminide aLloys: Science and Technology.

[4] Kamyshnykova K., Lapin J. (2018). Grain refinement of cast peritectic TiAl-based alloy by solid-state phase transformations. Kov. Mater..

[5] Lapin J., Kamyshnykova K. (2018). Processing, microstructure and mechanical properties of in-situ Ti_3_Al+TiAl matrix composite reinforced with Ti_2_AlC particles prepared by centrifugal casting. Intermetallics.

[6] Kim Y.W. (1998). Strength and ductility in TiAl alloys. Intermetallics.

[7] Wang Q., Chen R., Yang Y., Wu S., Guo J., Ding H., Su Y., Fu H. (2018). Effects of lamellar spacing on microstructural stability and creep properties in β-solidifying γ-TiAl alloy by directional solidification. Mater. Sci. Eng. A.

[8] Klein T., Usategui L., Rashkova B., Nó M.L., San Juan J., Clemens H., Mayer S. (2017). Mechanical behavior and related microstructural aspects of a nano-lamellar TiAl alloy at elevated temperatures. Acta. Mater..

[9] Liu Z.C., Lin J.P., Li S.J., Chen G.L. (2002). Effects of Nb and Al on the microstructures and mechanical properties of high Nb containing TiAl base alloys. Intermetallics.

[10] Lapin J., Pelachová T., Dománková M. (2018). Long-term creep behaviour of cast TiAl-Ta alloy. Intermetallics.

[11] Kastenhuber M., Klein T., Clemens H., Mayer S. (2018). Tailoring microstructure and chemical composition of advanced γ-TiAl based alloys for improved creep resistance. Intermetallics.

[12] Song L., Hu X., Wang L., Stark A., Lazurenko D., Lorenz U., Lin J., Pyczak F., Zhang T. (2019). Microstructure evolution and enhanced creep property of a high Nb containing TiAl alloy with carbon addition. J. Alloy. Compd..

[13] Lapin J. (2006). Comparative study of creep of cast Ti-46Al-2W-0.5Si and Ti-45Al-2W-0.6Si-0.7B alloys. Kov. Mater..

[14] Gabrisch H., Stark A., Schimansky F.P., Wang L., Schell N., Lorenz U., Pyczak F. (2013). Investigation of carbides in Ti-45Al-5Nb-xC alloys (0 ≤ x ≤ 1) by transmission electron microscopy and high energy-XRD. Intermetallics.

[15] Wang L., Oehring M., Lorenz U., Stark A., Pyczak F. (2018). New insights into perovskite-Ti_3_AlC precipitate splitting in a Ti-45Al-5Nb-0.75C alloy by transmission electron microscopy. Intermetallics.

[16] Cegan T., Szurman I. (2017). Thermal stability and precipitation strengthening of fully lamellar Ti-45Al-5Nb-0.2B-0.75C alloy. Kov. Mater..

[17] Lapin J., Klimová A., Gabalcová Z., Pelachová T., Bajana O., Štamborská M. (2017). Microstructure and mechanical properties of cast in-situ TiAl matrix composites reinforced with (Ti,Nb)_2_AlC particles. Mater. Des..

[18] Lapin J., Klimová A. (2019). Vacuum induction melting and casting of TiAl-based matrix in-situ composites reinforced by carbide particles using graphite crucibles and moulds. Vacuum.

[19] Lapin J., Štamborská M., Kamyshnykova K., Pelachová T., Klimová A., Bajana O. (2019). Room temperature mechanical behaviour of cast in-situ TiAl matrix composite reinforced with carbide particles. Intermetallics.

[20] Chen R., Fang H., Chen X., Su Y., Ding H., Guo J., Fu H. (2017). Formation of TiC/Ti_2_AlC and α_2_ + γ in in-situ TiAl composites with different solidification paths. Intermetallics.

[21] Chen R., Tan Y., Fang H., Luo L., Ding H., Su Y., Guo J., Fu H. (2018). Macro/microstructure evolution and mechanical properties of Ti33.3Al alloys by adding WC particles. Mater. Sci. Eng. A.

[22] Barsoum M.W., Ali M., El-Raghy T. (2000). Processing and characterization of Ti_2_AlC, Ti_2_AlN, and Ti_2_AlC_0.5_N_0.5_. Met. Mater. Trans. A.

[23] Klimová A., Lapin J. (2019). Effects of C and N additions on primary MAX phase particles in intermetallic Ti-Al-Nb-Mo matrix in-situ composites prepared by vacuum induction melting. Kov. Mater..

[24] Klimová A., Lapin J. (2019). Effect of Al content on microstructure of Ti-Al-Nb-C-Mo composites reinforced with carbide particles. Kov. Mater..

[25] Štamborská M., Lapin J., Bajana O. (2018). Effect of carbon on the room temperature compressive behaviour of Ti-44.5Al-8Nb-0.8Mo-xC alloys prepared by vacuum induction melting. Kov. Mater..

[26] Lapin J., Štamborská M., Pelachová T., Bajana O. (2018). Fracture behaviour of cast in-situ TiAl matrix composite reinforced with carbide particles. Mater. Sci. Eng. A.

[27] Lapin J., Pelachová T., Bajana O. (2019). High temperature deformation behaviour and microstructure of cast in-situ TiAl matrix composite reinforced with carbide particles. J. Alloy. Compd..

[28] Kastenhuber M., Rashkova B., Clemens H., Mayer S. (2015). Enhancement of creep properties and microstructural stability of intermetallic β-solidifying γ-TiAl based alloys. Intermetallics.

[29] Kastenhuber M., Rashkova B., Clemens H., Mayer S. (2017). Effect of microstructural instability on the creep resistance of an advanced intermetallic γ-TiAl based alloy. Intermetallics.

[30] Witusiewicz V.T., Hallstedt B., Bondar A.A., Hecht U., Sleptsov S.V., Velikanova T.Y. (2015). Thermodynamic description of the Al-C-Ti system. J. Alloy. Compd..

[31] Yang G., Yang X., Wang Y., Cheng L., Kou H., Liu Y., Li Y., Wang P., Ren W. (2020). Phase precipitation behavior of a quenched β-solidifying TiAl alloy with a fully-B2 microstructure during annealing at 800 °C. J. Alloy. Compd..

[32] Song L., Lin J., Li J. (2017). Phase transformation mechanisms in a quenched Ti-45Al-8.5Nb-0.2W-0.2B-0.02Y alloy after subsequent annealing at 800 °C. J. Alloy. Compd..

[33] Wang L., Zenk C., Stark A., Felfer P., Gabrisch H., Göken M., Lorenz U., Pyczak F. (2017). Morphology evolution of Ti_3_AlC carbide precipitates in high Nb containing TiAl alloys. Acta Mater..

[34] Witusiewicz V.T., Bondar A.A., Hecht U., Velikanova T.Y. (2009). The Al-B-Nb-Ti system. IV. Experimental study and thermodynamic re-evaluation of the binary Al-Nb and ternary Al-Nb-Ti systems. J. Alloy. Compd..

[35] Wang X., Yang J., Song L., Kou H., Li J., Fu H. (2017). Evolution of B2(ω) region in high-Nb containing TiAl alloy in intermediate temperature range. Intermetallics.

[36] Berglund I.S., Bryan Z.L., Manuel M.V. (2017). Kinetic modeling of the γ phase in Ti-Al-Nb alloys. J. Alloy. Compd..

[37] Karadge M., Gouma P.I. (2004). A structural aspect of α(α_2_) → lamellar α_2_ + γ transformation in γ-TiAl. Philos. Mag. Lett..

[38] Denquin A., Naka S. (1996). Phase transformation mechanisms involved in two-phase TiAl-based alloys-I. Lamellar structure formation. Acta Mater..

[39] Cabibbo M. (2020). Carbon content driven high temperature γ-α_2_ interface modifications and stability in Ti–46Al–4Nb intermetallic alloy. Intermetallics.

[40] Lapin J. (2003). Effect of lamellar structure on microhardness and yield stress of directionally solidified intermetallic Ti-46Al-2W-0.5Si alloy. J. Mater. Sci. Lett..

[41] Klein T., Schachermayer M., Mendez-Martin F., Schöberl T., Rashkova B., Clemens H., Mayer S. (2015). Carbon distribution in multi-phase γ-TiAl based alloys and its influence on mechanical properties and phase formation. Acta. Mater..

[42] Schloffer M., Rashkova B., Schöberl T., Schwaighofer E., Zhang Z., Clemens H., Mayer S. (2014). Evolution of the ωo phase in a β-stabilized multi-phase TiAl alloy and its effect on hardness. Acta. Mater..

[43] Velasco B., Gordo E., Hu L., Radovic M., Tsipas S.A. (2018). Influence of porosity on elastic properties of Ti_2_AlC and Ti_3_SiC_2_ MAX phase foams. J. Alloy. Compd..

[44] Kim H.Y., Maruyama K. (2001). Parallel twinning during creep deformation in soft orientation PST crystal of TiAl alloy. Acta. Mater..

[45] Du X.W., Zhu J., Kim Y.W. (2001). Microstructural characterization of creep cavitation in a fully-lamellar TiAl alloy. Intermetallics.

[46] Du X.W., Zhu J., Zhang X., Cheng Z.Y., Kim Y.W. (2000). Creep induced α_2_ → β phase transformation in a fully-lamellar TiAl alloy. Scr. Mater..

[47] Lapin J., Pelachová T., Dománková M. (2011). Creep behaviour of a new air-hardenable intermetallic Ti-46Al-8Ta alloy. Intermetallics.

[48] Lapin J., Nazmy M. (2004). Microstructure and creep properties of a cast intermetallic Ti-46Al-2W-0.5Si alloy for gas turbine applications. Mater. Sci. Eng. A.

[49] Schwaighofer E., Rashkova B., Clemens H., Stark A., Mayer S. (2014). Effect of carbon addition on solidification behavior, phase evolution and creep properties of an intermetallic β-stabilized γ-TiAl based alloy. Intermetallics.

[50] Maruyama K., Yamamoto R., Nakakuki H., Fujitsuna N. (1997). Effects of lamellar spacing, volume fraction and grain size on creep strength of fully lamellar TiAl alloys. Mater. Sci. Eng. A.

[51] Kim Y.W., Kim S.L. (2014). Effects of microstructure and C and Si additions on elevated temperature creep and fatigue of gamma TiAl alloys. Intermetallics.

[52] Kamyshnykova K., Lapin J. (2018). Vacuum induction melting and solidification of TiAl-based alloy in graphite crucibles. Vacuum.

[53] Lapin J., Ondrúš L., Nazmy M. (2002). Directional solidification of intermetallic Ti-46Al-2W-0.5Si alloy in alumina moulds. Intermetallics.

